# One session treatment (OST) is equivalent to multi‐session cognitive behavioral therapy (CBT) in children with specific phobias (ASPECT): results from a national non‐inferiority randomized controlled trial

**DOI:** 10.1111/jcpp.13665

**Published:** 2022-08-01

**Authors:** Barry Wright, Lucy Tindall, Alexander J. Scott, Ellen Lee, Cindy Cooper, Katie Biggs, Penny Bee, Han‐I Wang, Lina Gega, Emily Hayward, Kiera Solaiman, M. Dawn Teare, Thompson Davis, Jon Wilson, Karina Lovell, Dean McMillan, Amy Barr, Hannah Edwards, Jennifer Lomas, Chris Turtle, Steve Parrott, Catarina Teige, Tim Chater, Rebecca Hargate, Shezhad Ali, Sarah Parkinson, Simon Gilbody, David Marshall

**Affiliations:** ^1^ Leeds and York Partnership NHS Foundation Trust York UK; ^2^ Keele University Keele UK; ^3^ University of Sheffield Sheffield UK; ^4^ University of Manchester Manchester UK; ^5^ University of York York UK; ^6^ Louisiana State University Baton Rouge LA USA; ^7^ Norfolk and Suffolk NHS Foundation Trust Norwich UK

**Keywords:** Specific phobia, children and young people, one session treatment, cognitive behavioral therapy, randomized controlled trial, non‐inferiority

## Abstract

**Background:**

5%–10% children and young people (CYP) experience specific phobias that impact daily functioning. Cognitive Behaviour Therapy (CBT) is recommended but has limitations. One Session Treatment (OST), a briefer alternative incorporating CBT principles, has demonstrated efficacy. The Alleviating Specific Phobias Experienced by Children Trial (ASPECT) investigated the non‐inferiority of OST compared to multi‐session CBT for treating specific phobias in CYP.

**Methods:**

ASPECT was a pragmatic, multi‐center, non‐inferiority randomized controlled trial in 26 CAMHS sites, three voluntary agency services, and one university‐based CYP well‐being service. CYP aged 7–16 years with specific phobia were randomized to receive OST or CBT. Clinical non‐inferiority and a nested cost‐effectiveness evaluation was assessed 6‐months post‐randomization using the Behavioural Avoidance Task (BAT). Secondary outcome measures included the Anxiety Disorder Interview Schedule, Child Anxiety Impact Scale, Revised Children's Anxiety Depression Scale, goal‐based outcome measure, and EQ‐5DY and CHU‐9D, collected blind at baseline and six‐months.

**Results:**

268 CYPs were randomized to OST (*n* = 134) or CBT (*n* = 134). Mean BAT scores at 6 months were similar across groups in both intention‐to‐treat (ITT) and per‐protocol (PP) populations (CBT: 7.1 (ITT, *n* = 76), 7.4 (PP, *n* = 57), OST: 7.4 (ITT, *n* = 73), 7.6 (PP, *n* = 56), on the standardized scale‐adjusted mean difference for CBT compared to OST ‐0.123, 95% CI −0.449 to 0.202 (ITT), mean difference −0.204, 95% CI −0.579 to 0.171 (PP)). These findings were wholly below the standardized non‐inferiority limit of 0.4, suggesting that OST is non‐inferior to CBT. No between‐group differences were found on secondary outcomes. OST marginally decreased mean service use costs and maintained similar mean Quality Adjusted Life Years compared to CBT.

**Conclusions:**

One Session Treatment has similar clinical effectiveness to CBT for specific phobias in CYP and may be a cost‐saving alternative.

## Introduction

A specific phobia is an intense, enduring fear of a situation/object associated with anxiety symptoms, distress, and avoidance (American Psychiatric Association, 2013), estimated to affect 5% to 10% of children and young people (CYP). Specific phobias are associated with distress and interference with day‐to‐day activities (Ollendick & March, 2004), poorer quality of life (Comer et al., 2010), academic difficulties (Ialongo, Edelsohn, Werthamer‐Larsson, Crockett, & Kellam, 1995), and predict future mental health problems (Bittner et al., 2007; Lieb et al., 2016) including long‐term phobia (Meyer, Rumpf, Hapke, & John, 2004). Multi‐session Cognitive Behavioural Therapy (CBT) is the commonly used therapeutic approach to managing specific phobias in CYP and has a robust evidence base demonstrating efficacy (Hudson et al., 2015; Kendall & Hedtke, 2006). However, access to CBT is limited (Care Quality Commission, 2017), likely due to several factors. For example, multi‐session CBT is time‐consuming (Aschim, Lundevall, Martinsen, & Frich, 2011; Wiebe & Greiver, 2005), offered at great cost (Cavanagh, 2014; Shapiro, Cavanagh, & Lomas, 2003), and often requires highly trained therapists (Stallard, Myles, & Branson, 2014; van der Gaag, 2014). Moreover, multi‐session CBT takes several weeks and months to complete, which is burdensome for families accessing support (Ollendick, Ryan, Capriola‐Hall, Austin, & Fraire, 2018), and might be one reason for the relatively high drop‐out rate seen in CYP mental health services (De Haan, Boon, de Jong, Hoeve, & Vermeiren, 2013). These barriers to the provision of, and access to, multi‐session CBT suggest a need for briefer, cost‐effective treatments that retain the clinical benefits of CBT, while also improving access to therapy.

### One session treatment: a brief and effective treatment for specific phobia

One‐session treatment (OST) is a variant of CBT that uses many of the same techniques but does not require an extensive treatment period over several weeks and months. Instead, OST takes place over two sessions: (i) an initial assessment and planning session lasting around 1 hr, and (ii) a single exposure session lasting up to 3 hr. Furthermore, OST has a strong evidence base built largely over the last two decades, supporting its efficacy at improving specific phobias in CYP (Davis III, Ollendick, & Öst, 2019; Ollendick et al., 2015; Ryan, Strege, Oar, & Ollendick, 2017), therefore offering a clinically effective, briefer, and potentially more accessible treatment option to multi‐session CBT.

However, despite the clinical efficacy of OST, there remains substantial gaps in the evidence that prevent OST being delivered in routine clinical services. First, to our knowledge, no study has compared OST to multi‐session CBT. Consequently, it is unclear whether the clinical effects of OST stand up to the benefits of CBT. Second, extant research has tended to test OST in tightly controlled efficacy trials that do not reflect the participants, therapists, or environments seen in mental health services. Therefore, it is unclear how well OST would perform in a more pragmatic setting such as Child and Adolescent Mental Health Services (CAMHS). Finally, although the consolidated, brief format of OST lends itself to being a more cost‐effective tool than multisession CBT, to our knowledge, no study has performed an economic analysis to quantify any (potential) savings or evaluated the cost‐effectiveness of OST relative to CBT.

The present research The ‘Alleviating Specific Phobias Experienced by Children Trial’ (ASPECT) aims to address the gaps described above by investigating the clinical and cost‐effectiveness of OST vs. multi‐session CBT for specific phobias in CYP aged 7 to 16 years within ‘real‐world’ mental health services in the UK. The present research aimed to investigate the clinical non‐inferiority of OST compared to multi‐session CBT at a 6‐month follow‐up point in (i) reducing the severity of specific phobia as measured by the Behavioural Avoidance Task and (ii) improving wider quality of life outcomes for CYP. Additionally, we report the cost‐effectiveness of OST relative to multi‐session CBT (for a more detailed cost‐effectiveness analysis as part of ASPECT, see Wang et al., 2022).

## Methods

ASPECT was based upon an ethically approved protocol (Wright et al., 2018). This outlines the trial interventions, research processes, and statistical analyses in more depth and provides a rationale for the approaches adopted.

### Design

ASPECT was a pragmatic, multi‐center, parallel group non‐inferiority RCT, comparing the clinical and cost‐effectiveness of OST with multi‐session CBT. A nested qualitative study exploring the acceptability of OST to CYP, their parent/guardians, and therapists and a full economic evaluation are reported elsewhere (Hayward et al., 2022; Wang et al., 2022).

### Participants

Participants were recruited across thirteen sites in England, including twelve NHS Trusts (comprising a total of 26 CAMHS) and one University‐based CYP wellbeing service. Potential participants were identified by participating clinical services and referred to the ASPECT study team for screening. To be eligible for the study, participants were required to be aged 7 to 16 years (inclusive) who were experiencing a specific phobia as defined by DSM‐IV criteria (American Psychiatric Association, 2013). Specifically, participants were required to experience phobias characterized by (i) marked and out of proportion fear to a specific object or situation; (ii) exposure provokes immediate anxiety; (iii) the phobic situation(s) is avoided where possible; (iv) the avoidance or distress interferes with the person's routine or functioning; and (v) has been present for ≥ six‐months. CYPs were excluded from participation if (i) exposure therapy was not deemed to be an appropriate first‐line treatment (e.g., where a more severe difficulty took priority) and (ii) their phobic stimuli could not be safely presented or simulated during therapy (e.g., stinging insects). CYPs experiencing comorbid physical and/or mental health difficulties were not excluded, provided they met the eligibility criteria.

### Ethical considerations

After receiving study information, parent/guardians of interested participants expressed interest to the research team or *via* a clinician and provided fully informed written assent/consent. Health Research Authority and Research Ethics Committee approval from North East – York Ethics Research Committee [17/NE/0012] was received in February 2017.

### Procedure

Potential participants that were referred to ASPECT by participating recruitment sites were initially screened for eligibility by a Research Assistant over the telephone. Guardians of CYP with suspected phobias answered five ‘yes/no’ screening questions based on the Anxiety Disorder Interview Schedule (ADIS). Those eligible (answering ‘yes’ to all questions) were invited to a face‐to‐face baseline appointment with a Research Assistant where full informed consent and assent was taken from the Guardian and the CYP. After consent, baseline measurements were taken from both the Guardian and the CYP (see ‘Measurements’ section for all measurements). Participants were then remotely randomized by a Clinical Trials Unit to receive either CBT or OST (1:1) using an online system through the Trials Unit (see ‘Randomization’ section for full details). Participants were then referred to clinical services for their allocated therapy. All outcome measures (except the demographics survey) were repeated 6 months after randomization by a Research Assistant blind to group allocation.

### Measurements

#### Behavioral avoidance task (BAT)

The primary outcome was the BAT (Castagna, Davis, & Lilly, 2017), a widely used live behavioral outcome measure for assessing phobias in children. CYPs were exposed to their phobic stimulus over ten pre‐defined steps which gradually increased in difficulty. The number of steps taken was the main unit of measurement recorded for analysis, with more steps (i.e., a higher score) indicating less severe phobia. Alongside this, a measure of Subjective Units of Distress (SUDS) was also taken at the start of the BAT and at the last step completed, which ranged from 0 (no fear at all) to 8 (very, very much fear). All BATs were standardized, which included using the same/similar rooms and phobic stimuli, alongside the same Research Assistant. For some phobic stimuli, *in vivo* exposure was not ethically appropriate (e.g., needle injection, exposure to vomit). In these instances, phobic stimuli were simulated (e.g., using venipuncture practice arms to simulate injection, and simulated vomit).

#### The anxiety disorder interview schedule (ADIS)

The specific phobia subsection of the ADIS (Silverman & Albano, 1996) was administered by a trained Research Assistant to both the CYP and their parent/guardian independently. The ADIS is a semi‐structured interview that records information from the CYP and their Guardians about the type of specific phobia(s) experienced (e.g., animal, injection, heights), the degree of associated fear (ranging from 0 = not at all, to 8 = very, very much), whether the phobia impacts daily functioning (rated as ‘yes’ or ‘no’), and the degree to which the specific phobia impacts daily functioning (ranging from 0 = not at all, to 8 = very, very much). The ADIS interview was used to assign a Clinical Severity Rating (CSR) to both the CYP and Guardian perceptions of the specific phobia. In line with guidelines (Silverman & Albano, 1996), the higher value of the CSR's given by the CYP or the Guardian was taken for the final CSR, unless strong reason was provided to depart from this (e.g. clear evidence of unreliability of one score). Scores >4 were classed as meeting clinical thresholds as defined by the DSM (Silverman & Albano, 1996).

#### Child anxiety impact scale (CAIS)

The CAIS is a 27‐item parent and child self‐report questionnaire measuring anxiety‐related functional impairment across three sub‐domains; school activities, social life, and home/family life (Langley, Bergman, McCracken, & Piacentini, 2004). CYP and parent/guardians independently rate each statement using a 4‐point scale ranging from 0 (not at all) to 4 (very much).

#### The Revised Children's Anxiety and Depression Scale (RCADS)

The RCADS (Chorpita, Moffitt, & Gray, 2005), a 47‐item anxiety and depression measure, collects information across six sub‐domains; separation anxiety disorder, social phobia, generalized anxiety disorder, panic disorder, obsessive compulsive disorder, and major depressive disorder. Parents/guardians and CYP answer frequency statements for related items using a 4‐point scale ranging from 0 (never) to 3 (always). Threshold cut‐offs suggested by Chorpita et al. (2005) were used to aid interpretation.

#### EQ‐5D‐Y

The EQ‐5D‐Y (The EuroQol Group, 1990) is a widely used measure of CYP Health‐Related quality of life (HRQoL). Individuals classify their health on a 3‐point scale, 1 (no problems), 2 (some problems), and 3 (a lot of problems), over five dimensions (mobility, self‐care, usual activities, pain/discomfort, and anxiety/depression).

#### Child Health Utility 9D (CHU‐9D)

The CHU‐9D, a 9‐item questionnaire measuring CYP HRQoL (Stevens & Ratcliffe, 2012), requires CYP to select one of five sentences to describe their feelings regarding several constructs. The CHU‐9D allows the calculation of quality‐adjusted life years (QALYs) for cost‐utility analyses.

#### Goal‐based outcome measure

At baseline, CYP set three specific goals they would like to achieve as a result of treatment. Progress toward achieving the goal was assessed at the 6‐month follow‐up ranging from 0 (goal not met) to 10 (goal reached).

#### Resource utilization questionnaire

A resource use questionnaire, based upon previous measures focusing on CYP with mental health problems (Wright et al., 2014), was refined. Completed by parent/guardians, this collected all‐cause services data including community, mental health, and hospital‐based service use as well as days missed from school (CYP) or work (parent/guardian) in the preceding 6 months.

### Interventions

#### Cognitive Behavioral therapy (CBT)

CBT is the current gold‐standard approach to treating specific phobias and includes several elements that target cognitive and behavioral processes associated with specific phobia experience. CBT typically aims to help CYP recognize anxious feelings and bodily reactions to anxiety; understand interactions between thoughts, feelings, sensations, and the environment; confront the feared situation through exposure until anxiety reduces; and practice a range of anxiety management and coping strategies to enable progress. CBT is typically administered in hour‐long sessions delivered weekly, and although there is currently no recommended number of CBT sessions for specific phobias, CYP typically receive 6–12 sessions as part of usual care. ASPECT was a pragmatic trial aiming to reflect ‘real‐world’ delivery; therefore, therapists providing CBT as part of ASPECT were asked to deliver their service's usual multi‐session CBT approach.

#### One session treatment (OST)

OST comprises two treatment sessions – an initial 1‐hour functional assessment followed by a 3‐hour exposure session. OST is a variant of CBT and uses the same treatment techniques (e.g., graduated exposure, participant modeling, challenging unhelpful beliefs, and reinforcement). During the initial functional assessment session, the therapist works with the CYP to build rapport and develop a fear hierarchy (i.e., an ascending list of situations/experiences in order of perceived severity). In the subsequent exposure session, CYP work with the therapist to gradually move through their fear hierarchy, remaining at each step until subjective anxiety levels have decreased by ≥50%. Therapists delivering OST as part of the ASPECT trial were trained by experienced OST therapists based on the definitive textbook for OST (Davis, Ollendick, & Öst, 2012).

#### Therapist characteristics

Those involved in the delivery of interventions for ASPECT had a variety of roles including children's wellbeing practitioners (both school and CAMHS‐based), psychiatrists, nurses, and clinical psychologists. Most, 85% were based within CAMHS. Therapist characteristics are presented in Table S1.

### Randomization

Remote 1:1 randomization was conducted and stratified according to age (7‐11 years vs. 12–16 years) and phobia severity (ADIS CSR mild/moderate (scoring 4/5) vs. severe (scoring 6/7/8)) and restricted using randomly permuted blocks of size 4 and 6.

### Sample size

Prior meta‐analyses suggested that a standardized mean difference of around 0.8 on the BAT is clinically important (Jones, Jarvis, Lewis, & Ebbutt, 1996). Therefore, using the point estimate method, the non‐inferiority margin was set to be half of this at 0.4. The initial target sample size was 286 (143 per arm). An extension was requested when we faced COVID‐19 and other related recruitment challenges. Our observations at this point i.e., a correlation of 0.7 between baseline and six‐month primary outcome measures, an observed dropout rate of 27.3%, the finding that each therapist was treating five (instead of 15) CYP, and with a design effect of 1.04 (instead of the planned 1.14), the original sample size of 286 (143 per group), would have given us a power of 97.7%. Based on these observations, a total of 246 participants (123 per group) were required to preserve a power of ~90% for a one‐sided 2.5% test with a standardized non‐inferiority margin of 0.4. This was approved by the Data Monitoring and Ethics Committee (DMEC), Trial Steering Committee (TSC) and the funder.

### Statistical analysis

Scores on the continuous primary and secondary outcomes were compared between groups using mixed‐effects linear regression, with exchangeable correlation allowing for clustering of outcomes within therapist. Analysis controlled for baseline scores and stratifying variables (age, phobia severity). Secondary binary outcomes were analyzed using a mixed‐effects logistic regression model adjusted for age, site, phobia severity, and therapist as the random effect. For the primary outcome, the standardized mean difference was calculated using Hedges correction factor and the confidence interval using the true standard error (Goulet‐Pelletier & Cousineau, 2018), and the null hypothesis of inferiority would have been rejected if the lower limit of the two‐sided 95% confidence interval (CI) for the standardized mean difference was wholly below 0.4 (the range of clinical non‐inferiority).

To conclude non‐inferiority of OST, we required both the intention‐to‐treat (ITT) and the PP analyses to reject the null hypothesis of non‐inferiority. To be considered PP CYP randomized to OST had to have attended one assessment session, one main exposure session (with an optional extra session), and treatment needed to include an assessment, fear hierarchy development, and exposure. Any CYP attending more than the three outlined sessions prior to 6‐months follow‐up was not considered PP. CYP randomized to CBT were defined as PP if they had attended ≥ four sessions.

#### Fidelity assessment

Where possible, treatment sessions were audio‐recorded, and a random sample were independently rated for fidelity by clinical members of the research team using the One Session Treatment Rating Scale (OST‐RS, Ollendick et al., 2015) or the Cognitive Behaviour Therapy Scale for CYP (CBTS‐CYP, Stallard et al., 2014). A sample of 15 treatment sessions was selected for fidelity assessment.

#### Health economics analysis

Although a full economic analysis of OST vs. multi session CBT as part of ASPECT is reported elsewhere (Wang et al., 2022), we report the main cost‐effectiveness findings here. In brief, an economic analysis was conducted to evaluate the cost‐effectiveness (expressed as the incremental cost‐effectiveness ratio (ICER)) of OST. Both resource use from the NHS and personal social services perspective and quality adjusted life years (QALYs) measured by EQ‐5D‐Y were collected at baseline and at 6‐month follow‐up. Regression models controlled for baseline differences in cost and utility were used to compare mean costs and QALYs, and a non‐parametric bootstrapping with 5,000 iterations was conducted to take uncertainty into consideration. The bootstrapped results were presented in the conventional form of a cost‐effectiveness acceptability curve (CEAC), which resented the probability of the intervention being cost‐effective over a range of willingness‐to‐pay thresholds per QALY.

## Results

### Recruitment

**Figure 1:**
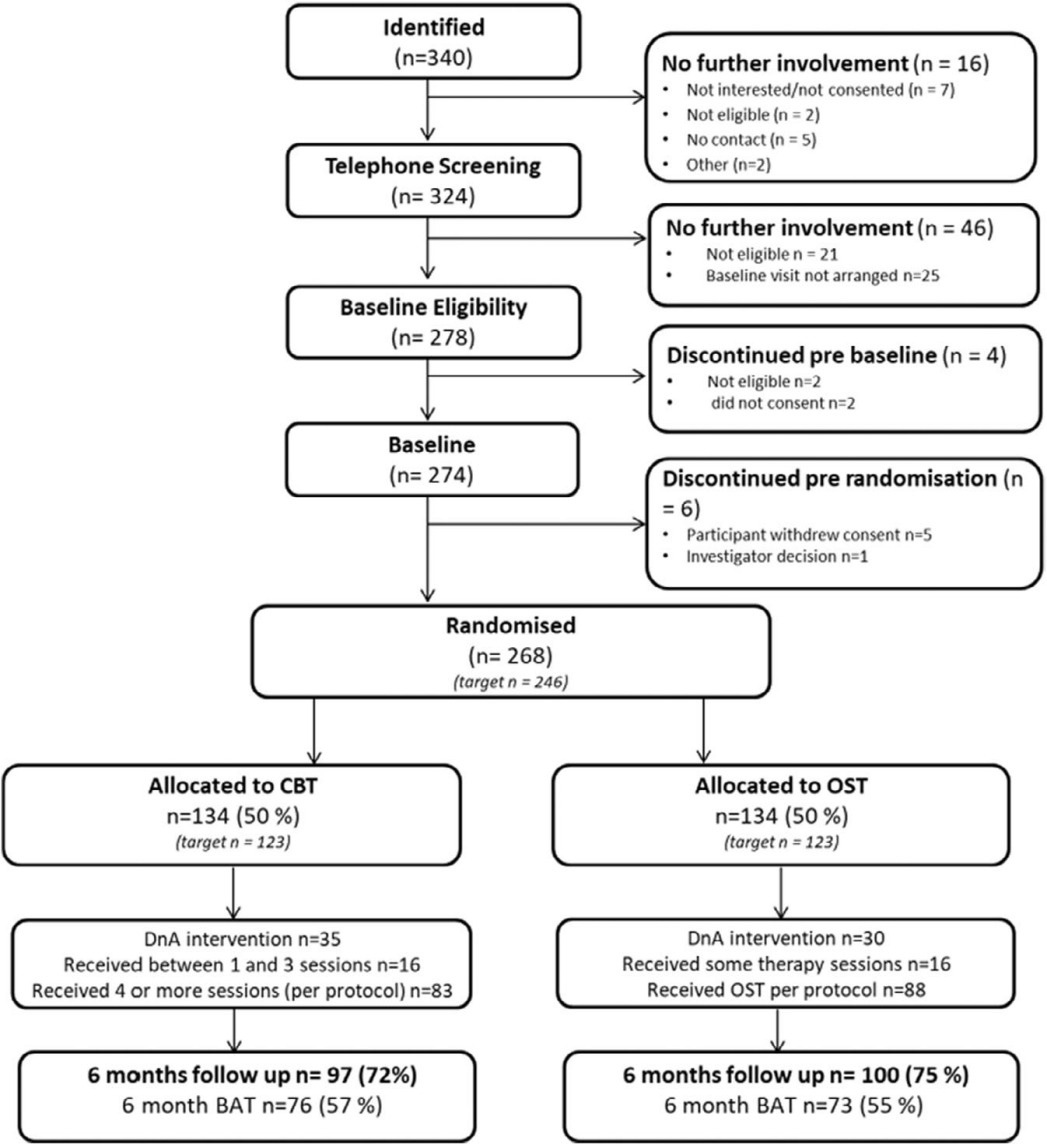
CONSORT diagram showing participant flow

Participant flow is presented in Figure 1. Over 31 months (June 2017 to January 2020), *n* = 274 CYP were recruited, with *n* = 268 randomized to CBT (*n* = 134) or OST (*n* = 134). *n* = 197 CYP provided six‐month follow‐up data, with *n* = 149 (56%) completing the primary outcome measure (BAT) as part of this. Due to the COVID‐19 pandemic, government restrictions meant that some participants (*n* = 48) had 6‐month follow‐up data collected remotely and therefore were unable to complete the primary outcome measure (i.e., the BAT which requires face‐to‐face exposure). Reasons for withdrawal post randomization included CYP withdrawing consent (*n* = 26), CYP being lost to follow‐up (*n* = 36), or an investigator decision (*n* = 9).

### Sample characteristics

Table S2 describes CYP and parent/guardian baseline characteristics. Of those randomized, participant mean age was 12 years, 62% were female, and 96% White British. 90% parents/guardians were female. The most common phobias were vomiting (29%), dogs (21%), and receiving injections (17%). Baseline assessment results were comparable across groups. All participants met diagnostic criteria for specific phobia as a requirement of trial participation, and most participants were assessed as having severe phobia (ADIS median (IQR): 8 (7–8)), reflecting the high threshold for CAMHS entry. The median number of BAT steps completed at baseline across both groups was three steps out of a possible ten. The RCADS total anxiety median (IQR) was 30.0 (17.0–49.0), with *N* = 24 (9%) of children scoring at least 65, suggesting borderline clinical anxiety.

### Treatment summaries

Excluding participants that received no treatment, and those randomized to CBT received an average of 6.1 treatment hours over 5.9 sessions; (5.4 h if sessions after the six‐month follow‐up are excluded). OST participants had an average of 2.2 sessions conducted over 3.8 h with 63% receiving treatment PP, 6% receiving too much treatment (too long/too many sessions), and 31% not attending any treatment sessions within 6 months. 57% of CBT participants received treatment PP (≥ four sessions), 16% received between one and three sessions, and 27% received no treatment. For some CYP, this was related to COVID‐19, although the exact figure for this was unavailable.

#### Primary outcome

**Figure 2:**
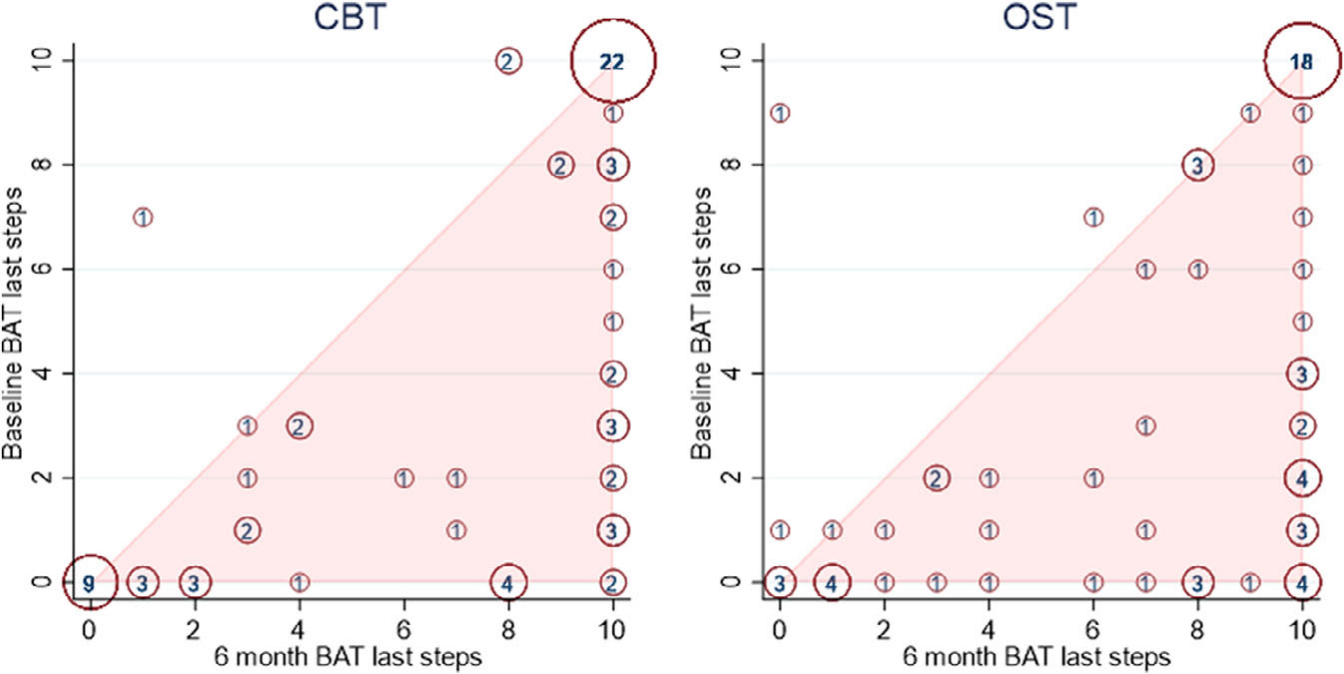
Bubble plot of BAT last steps by the treatment group for the intention‐to‐treat population [Color figure can be viewed at wileyonlinelibrary.com]

**Figure 3:**
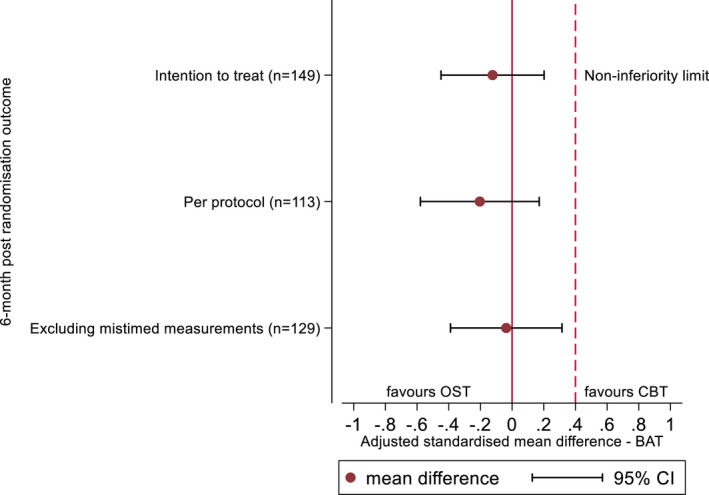
Primary and sensitivity analyses of BAT steps between groups on the standardised scale [Color figure can be viewed at wileyonlinelibrary.com]

Analysis of the primary outcome (i.e., the BAT) suggested that OST was clinically non‐inferior to multi‐session CBT. From baseline to six‐months follow‐up, improvements were seen in the number of BAT steps taken in both the ITT and PP populations, with a higher number of CYP attaining step 10 at 6 months compared to baseline (Figure 2). A marginally larger improvement was seen for the PP group in both treatment arms. Progression in the number of BAT steps taken was comparable across groups at 6 months (CBT 7.1 (ITT) and 7.4 (PP); OST 7.4 (ITT) and 7.6 (PP); adjusted mean difference for CBT compared to OST −0.46, 95% CI −1.43 to 0.51 for ITT, mean difference − 0.73, 95% CI ‐1.83 to 0.37 for PP; Table 1) compared to baseline (3). As the standardized mean difference 95% CIs were below the non‐inferiority limit (0.4; Figure 3), the results provide evidence for OST as non‐inferior to CBT. A similar proportion in each group was assessed at the 6‐month follow‐up as still having a phobia diagnosis, for both ITT and PP populations (see Table 2).

**Table 1:** Comparison of mean 6‐month BAT last steps by treatment groups (*n* = 268)

Outcome ‐ BAT six‐months	Treatment group							
	CBT		OST		Raw scale^a^		Standardised^b^	
	n	Mean (SD)	n	Mean (SD)	Adjusted mean difference	95% CI	Adjusted mean difference	95% CI
Intention‐to‐treat	76	7.1 (3.9)	73	7.4 (3.6)	−0.46	−1.43 to 0.51	−0.123	−0.449 to 0.202
Per‐protocol	57	7.4 (3.7)	56	7.6 (3.3)	−0.73	−1.83 to 0.37	−0.204	−0.579 to 0.171
Excluding mistimed measurements^c^	61	7.5 (3.7)	68	7.4 (3.6)	−0.13	−1.27 to 1.00	−0.037	−0.389 to 0.315
Multiple imputation^d^	134	6.9 (3.9)	134	7.5 (3.8)	−0.1	−1.1 to 0.8		
CACE ‐ CBT pp^e^					−0.51	−1.76 to 0.73		
CACE ‐ OST pp^e^					−0.48	−1.63 to 0.67		

BAT is measured on a 0 (no steps) to 10 (all steps completed) scale. A positive difference means the CBT group completed more steps at 6 months than the OST group.

^a^ Adjusted for baseline BAT score, age, phobia severity (ADIS CRS), site as fixed effects, and therapist as a random effect.

^b^ Standardized mean difference and confidence interval calculated using the Hedges correction factor and true standard error outlined in section 2.1.12.

^c^ Mistimed measures are BAT follow‐up taken outside 4 weeks before to 6 weeks after six‐month post randomization.

^d^ Multiple imputation using chained equations (regression) based on 100 imputed data sets with baseline BAT, age, ethnicity, treatment preference ADIS CSR sex site, and 6 month CAIS, RCADS total anxiety, and EQ‐5D‐Y as covariates.

^e^ Complier Average Causal Effect (CACE) using two stage least squares regression with age, site, ADIS composite, EQ‐5D‐Y and BAT at baseline as covariates and standard errors that allow for intragroup correlation by therapist. All other analyses use a mixed‐effects regression model.

**Table 2:** Comparison of proportion with specific phobia at 6 months

Outcome	Treatment group			
	CBT	OST	Adjusted^a^	
	*n* (%)	*n* (%)	OR	95% CI
Intention to treat				
Specific phobia (ADIS CSR > =4)	71 (73%)	73 (73%)	0.96	0.45 to 2.03
Per‐protocol				
Specific phobia (ADIS CSR > =4)	46 (68%)	49 (66%)	1.41	0.60 to 3.32

^a^ Adjusted for age, site and baseline ADIS CSR as fixed effects and therapist as a random effect.

#### Sensitivity analyses of the primary outcome

Three pre‐planned sensitivity analyses were conducted and displayed alongside the ITT and PP analysis in Figure 3. The first of these used multiple imputation and chained equations (where the uncertainty surrounding missing data is addressed by creating different plausible imputed data sets and combining the results). The second excluded participants with data collected more than 4 weeks pre and 6 weeks post the 6‐month follow‐up date. The final sensitivity analysis in relation to OST and CBT compliance was conducted via Complier Average Causal Effect (CACE) analysis (Angrist, Imbens, & Rubin, 1996). Receipt of treatment was regressed on the random treatment allocation and baseline covariates, generating a prediction of the receipt of treatment. The primary analysis model was fitted after replacing treatment with the prediction. CACE analysis was conducted for both CBT and OST. All sensitivity analyses found that the 95% CIs remained wholly below the non‐inferiority limit, confirming the robustness of the primary result.

#### Secondary outcomes

Secondary outcomes were comparable to the primary outcome for both groups (see Table S3 for more details). Some point estimates were slightly in favor of CBT and others slightly in favor of OST, but all confidence intervals crossed zero.

#### Safety

Four serious adverse events were recorded during the trial (all inpatient hospitalizations) and were assessed by the site PI as unrelated to the trial treatments.

#### Fidelity assessment results

Seventy treatment sessions were recorded in total, with 39 CBT and four OST sessions were available to assess fidelity. Session 1 in OST and sessions 1 and 2 in CBT were excluded from fidelity assessment as these are wholly or partly assessment sessions and are not expected to include the whole range of the intervention. Eleven CBT‐recorded sessions were randomly selected, and all available OST recorded sessions were selected for fidelity assessment. All sessions were rated above satisfactory, using criteria from the validated fidelity assessments (OST; M = 56.5 (range 55–58), CBT; M = 70.3 (range 55–83)), indicating higher therapist competencies in the CBT‐assessed sessions. Using guidelines suggested by Borrelli (2011), 8/15 sessions (53%) were classified as high fidelity and 7/15 (47%) sessions as moderate, with none at low fidelity.

### Health economics results

**Figure 4:**
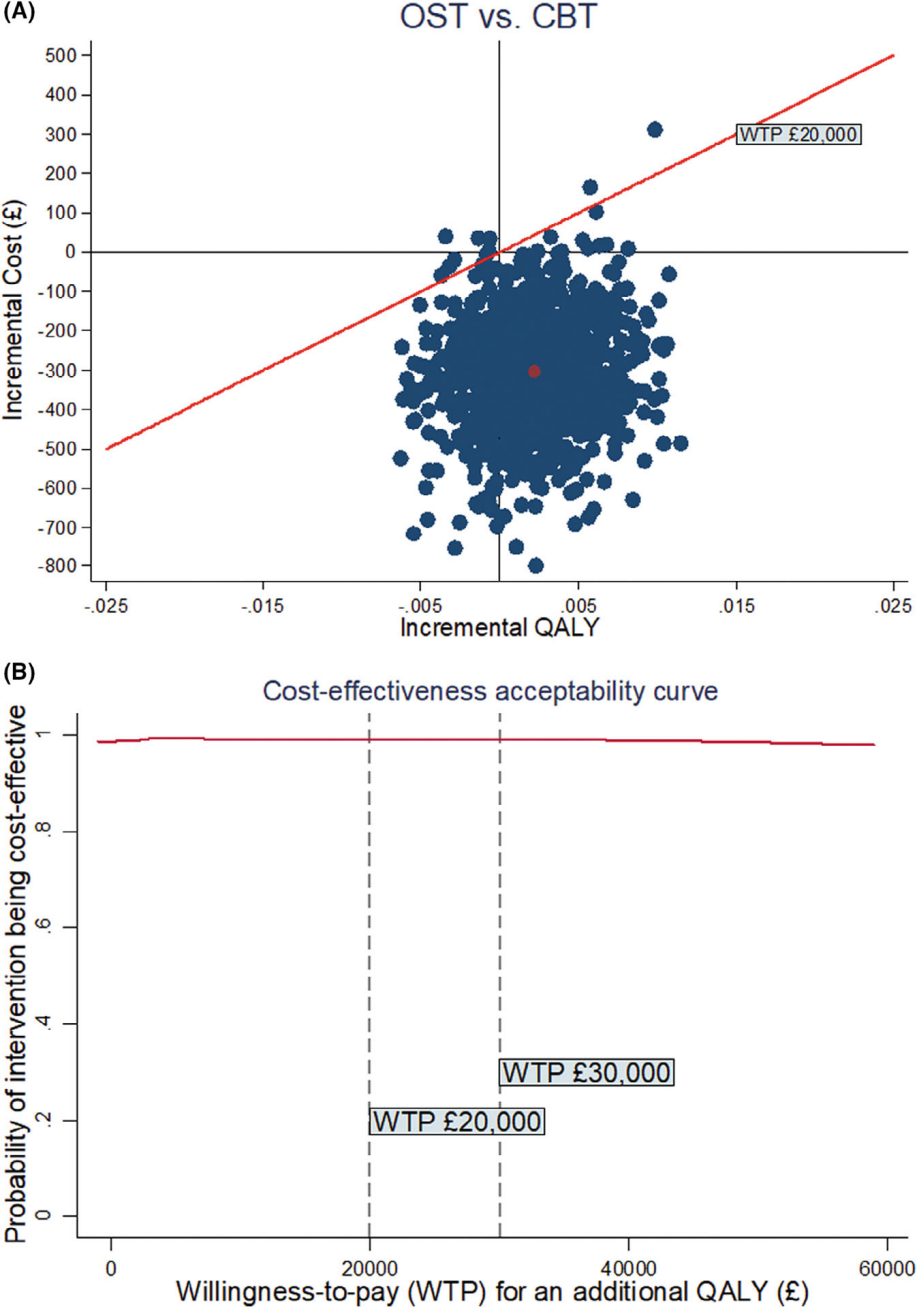
Cost‐effectiveness plane and CEAC (QALY) measured by EQ‐5D‐Y and costs estimated from a NHS/PSS perspective [Color figure can be viewed at wileyonlinelibrary.com]

After multiple imputation and bootstrapping, on average, CYP randomized to OST incurred less costs (incremental cost: ‐£303 (95% CI ‐£599 to ‐£29) and maintained similar improvement in QALYs +0.002 (95% CI −0.004 to 0.008)). The Cost Effectiveness Acceptability Curve (CEAC) shows that the probability of OST being cost‐effective was over 97% across all WTP thresholds, suggesting OST is likely to be cost‐saving compared to CBT (Figure 4). The full health economic findings are reported elsewhere (Wang et al., 2022).

## Discussion

Using prospectively set non‐inferiority parameters, OST is shown to be non‐inferior in clinical effectiveness to multi‐session CBT when treating specific phobias in CYP. The number of steps completed on the BAT (our primary outcome) and the percentage of diagnosis‐free participants at follow‐up were similar for both OST and CBT. The non‐inferiority conclusion was corroborated by our secondary outcomes (SUDS, CAIS, goal‐based outcomes and RCADS).

The value of ASPECT is that it is the only fully powered RCT that has compared OST with another active therapy across a range of phobias. The findings of an earlier small RCT (Flatt & King, 2010) with 43 participants across three arms (OST, cognitive therapy, and waiting list) are uninterpretable due to the small sample. Two earlier RCTs by Ollendick and colleagues compared (a) OST against psychological placebo and waiting list (Ollendick et al., 2009) that found OST to be superior to education support and waiting list and (b) different versions of OST (with or without parental involvement, Ollendick et al., 2015). There are, therefore, no similar previous studies for comparison with ours.

We cannot tell whether the number of steps completed on the BAT and the percentage of diagnosis‐free participants at follow‐up would be higher if COVID‐19 had not affected treatment completion. Absolute rates at follow‐up should be considered in the context of our sample having higher severity at baseline and including more complex presentations, such as blood–injury–injection phobia and young people with low motivation, who were excluded by other studies (e.g. Ollendick et al., 2009). We also determined diagnosis conservatively by choosing the higher score between parent and child (as done by Evans, Thirlwall, Cooper, & Creswell, 2017), rather than averaging the scores (as in Ollendick et al., 2009), which could have deflated the absolute numbers of “diagnosis‐free” participants at follow‐up.

Findings from the qualitative and health economics analyses are presented elsewhere and demonstrated good acceptability and satisfaction with OST from the perspectives of CYP, parents/guardians and clinicians, and also marginally decreased service use costs with similar QALYs, which will be of interest to commissioners.

### Strengths and limitations

#### Strengths

ASPECT is the first RCT comparing the clinical and cost‐effectiveness of OST to multi‐session CBT for CYP with specific phobias. Unlike other previous studies conducted in tightly controlled research settings (Ollendick et al., 2009, 2015; Öst, Svensson, Hellström, & Lindwall, 2001), ASPECT's pragmatic design allowed the effectiveness of OST and CBT to be compared in real‐world clinical settings. Furthermore, by imposing few exclusions, many phobia types were represented in ASPECT allowing broad comparisons of OST and CBT to be made, unlike previous studies (Ollendick et al., 2009) where BII phobias were excluded. BII phobias are particularly important because they lead to vaccination refusals and get in the way of medical screening and dental check‐ups.

ASPECT employed several methodological mechanisms to reduce bias including remote randomization, blinding of researchers collecting outcome measures, and adherence to a prospectively planned protocol, alongside statistical and health economics analysis plans, conducting treatment fidelity assessments and receiving project oversight from an independent Trial Steering Committee and Data Monitoring Ethics Committee. Including the BAT alongside self‐reported questionnaires allowed for a comprehensive and objective measure of CYP phobia.

#### Limitations

The COVID‐19 pandemic significantly affected therapy delivery and completion of follow‐up assessments; 48 CYP did not complete the primary outcome measure (BAT) at follow‐up. 37% (*n* = 98) CYP completed their six‐month follow‐up assessments having received no or incomplete treatment.

### Implications and recommendations

Access to training for community or school‐based child mental health treatments has become more readily available to UK clinicians (Department of Health and Social Care and Department for Education, 2017). As this includes training to deliver OST, ASPECT was timely in providing evidence for OST being as effective as CBT for CYP with specific phobias. Our study shows OST has a place in the armory of possible available treatments for specific phobias. For young people who do not engage or benefit from OST and CBT, further research can help us understand “why” and subsequently enable us to develop and evaluate new strategies for maximizing therapy gains for a greater number of young people.

## Conclusions

To date, ASPECT is the only fully powered RCT to compare the clinical and cost‐effectiveness of OST relative to multi‐session CBT. Our findings show that OST is as clinically effective as multi‐session CBT for specific phobia in CYP and is likely to be more cost‐effective. Future work should focus on developing service specifications, training, and care pathways to ensure that OST can be readily available as part of routine care for severe and debilitating phobias in children and young people.

## Supporting information


**Table S1.** Characteristics of the therapists delivering OST and CBT in the trial (*n* = 85).
**Table S2.** Baseline characteristics by the randomized group for all randomized participants and parents/guardians of all randomized participants (*n* = 268).
**Table S3.** Comparison of mean six‐month secondary assessments by treatment group (*N* = 197).
