# Supplementary material for: One session treatment (OST) is equivalent to multi‐session cognitive behavioral therapy (CBT) in children with specific phobias (ASPECT): results from a national non‐inferiority randomized controlled trial
Source: J Child Psychol Psychiatry. 2022 Aug 1;64(1):39–49. doi: 10.1111/jcpp.13665 (PMC10087411; doi:10.1111/jcpp.13665)
Supplement: Supplementary file 1 — Table S1. Characteristics of the therapists delivering OST and CBT in the trial (n = 85). Table S2. Baseline characteristics by the randomized group for all randomized participants and parents/guardians of all randomized participants (n = 268). Table S3. Comparison of mean six‐month secondary assessments by treatment group (N = 197). [file JCPP-64-39-s001.docx]

Supporting Information

**Table S1.** *Characteristics of the therapists delivering OST and CBT in the trial (n=85)*

| Characteristic | Summary |
| --- | --- |
|  | (N=85) |
| Number of years experience* | |
| N | 53 |
| Mean (SD) | 8 (8) |
| Median (IQR) | 5 (2, 12) |
| Min., Max. | 0, 32 |
|  |  |
| Treatment preference, n(%) | |
| No preference | 17 (35%) |
| Preference for CBT | 14 (29%) |
| Preference for OST | 17 (35%) |
|  |  |
| Organisation, n(%) | |
| CAMHS | 45 (85%) |
| Local Education Authority (School-based service) | 4 (8%) |
| Third sector voluntary agency | 2 (4%) |
| University-based children and young people's wellbeing service | 2 (4%) |
|  |  |
| Role, n(%) | |
| Assistant/Trainee Psychologist | 6 (9%) |
| CBT therapist | 6 (9%) |
| Clinical psychologist | 9 (14%) |
| Counsellor | 2 (3%) |
| Medical doctor | 2 (3%) |
| Nurse | 3 (5%) |
| Other | 14 (22%) |
| Psychiatrist | 1 (2%) |
| Trainee CBT therapist | 3 (5%) |
| Wellbeing practitioner (CAMHS-based) | 12 (19%) |
| Wellbeing practitioner (Schools-based) | 6 (9%) |
|  |  |
| Qualification | |
| GBC | 3 (4%) |
| BACP | 2 (2%) |
| BPS DClinPsy | 8 (9%) |
| BABCP psychotherapist accreditation | 9 (11%) |
| BABCP trainer accreditation | 3 (4%) |
| BABCP accredited wellbeing practitioner | 2 (2%) |
| BABCP accredited supervisor | 3 (4%) |
| MBBS | 3 (4%) |
| MRCPsych | 2 (2%) |
| Counselling Diploma | 2 (2%) |
| Diploma in CBT | 17 (20%) |
| Certificate in CBT | 8 (9%) |
| CYP IAPT | 17 (20%) |
| PGCE | 2 (2%) |
| Other associate degree | 3 (4%) |
| Other bachelor's degree | 11 (13%) |
| Other graduate diploma | 9 (11%) |
| Other masters degree | 3 (4%) |
| Other doctorate | 1 (1%) |

* Min. minimum, Max. maximum Delivering psychological interventions/therapies with children and young people

Table S2. *Baseline characteristics by randomised group for all randomised participants and parents/guardians of all randomised participants (n=268)*

| **Characteristic** | **Randomised participants** | | | **Parents and guardians** | | |
| --- | --- | --- | --- | --- | --- | --- |
|  | **CBT** | **OST** | **All** | **CBT** | **OST** | **All** |
| **Age** |  |  |  |  |  |  |
| N (%) | 134 (100%) | 134 (100%) | 268 (100%) | 132 (99%) | 133 (99%) | 265 (99%) |
| Mean (SD) | 11.8 (2.6) | 11.8 (2.6) | 11.8 (2.6) | 41.3 (6.4) | 42.9 (7.2) | 42.1 (6.9) |
| Median (IQR) | 12.0 (10.0, 14.0) | 12.0 (10.0, 14.0) | 12.0 (10.0, 14.0) | 42.0 (37.0, 46.0) | 43.0 (37.0, 48.0) | 42.0 (37.0, 47.0) |
| **Sex** |  |  |  |  |  |  |
| Boy | 53 (40%) | 48 (36%) | 101 (38%) | 15 (11%) | 12 (9%) | 27 (10%) |
| Girl | 81 (60%) | 86 (64%) | 167 (62%) | 119 (89%) | 122 (91%) | 241 (90%) |
|  |  |  |  |  |  |  |
| **Ethnicity** |  |  |  |  |  |  |
| English / Welsh / Scottish / Northern Irish / British | 129 (96%) | 127 (95%) | 256 (96%) | 127 (95%) | 126 (94%) | 253 (94%) |
| Irish | 0 (0%) | 1 (1%) | 1 (0%) | 1 (1%) | 0 (0%) | 1 (0%) |
| Any other White background | 0 (0%) | 1 (1%) | 1 (0%) | 4 (3%) | 4 (3%) | 8 (3%) |
| Pakistani | 1 (1%) | 2 (1%) | 3 (1%) | 1 (1%) | 2 (1%) | 3 (1%) |
| White and Black Caribbean | 1 (1%) | 0 (0%) | 1 (0%) | 0 (0%) | 1 (1%) | 1 (0%) |
| White and Black African | 2 (1%) | 0 (0%) | 2 (1%) | 1 (1%) | 0 (0%) | 1 (0%) |
| White and Asian | 0 (0%) | 2 (1%) | 2 (1%) | 0 (0%) | 1 (1%) | 1 (0%) |
| Any other mixed / multiple ethnic background | 0 (0%) | 1 (1%) | 1 (0%) | 127 (95%) | 126 (94%) | 253 (94%) |
| Prefer not to say | 1 (1%) | 0 (0%) | 1 (0%) | 1 (1%) | 0 (0%) | 1 (0%) |
|  |  |  |  |  |  |  |
| **Principal diagnosis (ADIS)** |  |  |  |  |  |  |
| Spiders | 5 (4%) | 6 (4%) | 11 (4%) |  |  |  |
| Dogs | 30 (22%) | 26 (19%) | 56 (21%) |  |  |  |
| Bees / Insects | 5 (4%) | 6 (4%) | 11 (4%) |  |  |  |
| Other animals | 1 (1%) | 1 (1%) | 2 (1%) |  |  |  |
| Heights | 1 (1%) | 2 (1%) | 3 (1%) |  |  |  |
| Darkness | 2 (1%) | 0 (0%) | 2 (1%) |  |  |  |
| Getting injections | 23 (17%) | 23 (17%) | 46 (17%) |  |  |  |
| Having blood tests | 7 (5%) | 8 (6%) | 15 (6%) |  |  |  |
| Seeing blood from a cut or scrape | 1 (1%) | 5 (4%) | 6 (2%) |  |  |  |
| Lifts or small enclosed places | 0 (0%) | 2 (1%) | 2 (1%) |  |  |  |
| Doctors / Dentists | 1 (1%) | 2 (1%) | 3 (1%) |  |  |  |
| Vomiting | 41 (31%) | 36 (27%) | 77 (29%) |  |  |  |
| Costumed characters | 5 (4%) | 1 (1%) | 6 (2%) |  |  |  |
| Choking | 0 (0%) | 4 (3%) | 4 (1%) |  |  |  |
| Food / Eating | 3 (2%) | 5 (4%) | 8 (3%) |  |  |  |
| Other | 9 (7%) | 7 (5%) | 16 (6%) |  |  |  |
|  |  |  |  |  |  |  |
| **ADIS CSR** |  |  |  |  |  |  |
| N (%) | 134 (100%) | 134 (100%) | 268 (100%) |  |  |  |
| Mean (SD) | 7.5 (0.9) | 7.6 (0.9) | 7.5 (0.9) |  |  |  |
| Median (IQR) | 8.0 (7.0, 8.0) | 8.0 (7.0, 8.0) | 8.0 (7.0, 8.0) |  |  |  |
|  |  |  |  |  |  |  |
| **BAT steps** |  |  |  |  |  |  |
| N (%) | 134 (100%) | 133 (99%) | 267 (100%) |  |  |  |
| Mean (SD) | 4.1 (4.2) | 4.8 (4.2) | 4.4 (4.2) |  |  |  |
| Median (IQR) | 2.0 (0.0, 9.0) | 4.0 (0.0, 10.0) | 3.0 (0.0, 10.0) |  |  |  |
|  |  |  |  |  |  |  |
| **Change in SUDS** |  |  |  |  |  |  |
| N (%) | 121 (90%) | 120 (90%) | 241 (90%) |  |  |  |
| Mean (SD) | -0.3 (2.3) | -0.1 (1.9) | -0.2 (2.1) |  |  |  |
| Median (IQR) | 0.0 (-1.0, 0.0) | 0.0 (-1.0, 1.0) | 0.0 (-1.0, 1.0) |  |  |  |
|  |  |  |  |  |  |  |
| **CAIS** |  |  |  |  |  |  |
| N (%) | 130 (97%) | 133 (99%) | 263 (98%) | 127 (95%) | 129 (96%) | 256 (96%) |
| Mean (SD) | 17.8 (13.6) | 18.1 (14.1) | 17.9 (13.8) | 20.2 (16.3) | 21.9 (16.7) | 21.1 (16.5) |
| Median (IQR) | 15.0 (8.0, 24.0) | 14.0 (7.0, 26.0) | 15.0 (7.0, 25.0) | 16.0 (6.0, 30.0) | 17.0 (8.0, 33.0) | 16.0 (8.0, 32.0) |
|  |  |  |  |  |  |  |
| **CHU-9D** |  |  |  |  |  |  |
| N (%) | 133 (99%) | 132 (99%) | 265 (99%) |  |  |  |
| Mean (SD) | 0.9 (0.1) | 0.8 (0.1) | 0.9 (0.1) |  |  |  |
| Median (IQR) | 0.9 (0.8, 0.9) | 0.9 (0.8, 0.9) | 0.9 (0.8, 0.9) |  |  |  |
|  |  |  |  |  |  |  |
|  |  |  |  |  |  |  |
| **EQ-5D-Y** |  |  |  |  |  |  |
| N (%) | 134 (100%) | 133 (99%) | 267 (100%) |  |  |  |
| Mean (SD) | 0.741 (0.276) | 0.748 (0.258) | 0.745 (0.266) |  |  |  |
| Median (IQR) | 0.812 (0.656, 1.000) | 0.812 (0.689, 0.883) | 0.812 (0.689, 1.000) |  |  |  |
|  |  |  |  |  |  |  |
| **EQ-5D VAS** |  |  |  |  |  |  |
| N (%) | 134 (100%) | 133 (99%) | 267 (100%) |  |  |  |
| Mean (SD) | 79.1 (19.6) | 77.7 (18.6) | 78.4 (19.1) |  |  |  |
| Median (IQR) | 85.0 (70.0, 95.0) | 80.0 (65.0, 95.0) | 80.0 (70.0, 95.0) |  |  |  |
|  |  |  |  |  |  |  |
| **Goal based outcome - goal 1** |  |  |  |  |  |  |
| N (%) | 134 (100%) | 134 (100%) | 268 (100%) |  |  |  |
| Mean (SD) | 1.7 (2.0) | 1.9 (1.9) | 1.8 (2.0) |  |  |  |
| Median (IQR) | 1.0 (0.0, 3.0) | 1.5 (0.0, 3.0) | 1.0 (0.0, 3.0) |  |  |  |
|  |  |  |  |  |  |  |
| **Goal based outcome - goal 2** |  |  |  |  |  |  |
| N (%) | 127 (95%) | 129 (96%) | 256 (96%) |  |  |  |
| Mean (SD) | 1.9 (2.1) | 2.1 (1.9) | 2.0 (2.0) |  |  |  |
| Median (IQR) | 1.0 (0.0, 3.0) | 2.0 (0.0, 4.0) | 2.0 (0.0, 3.5) |  |  |  |
|  |  |  |  |  |  |  |
| **Goal based outcome - goal 3** |  |  |  |  |  |  |
| N (%) | 89 (66%) | 92 (69%) | 181 (68%) |  |  |  |
| Mean (SD) | 2.0 (2.0) | 1.9 (1.8) | 1.9 (1.9) |  |  |  |
| Median (IQR) | 1.0 (0.0, 3.0) | 2.0 (0.0, 3.0) | 2.0 (0.0, 3.0) |  |  |  |
|  |  |  |  |  |  |  |
| **RCADS social phobia score** |  |  |  |  |  |  |
| N (%) | 134 (100%) | 133 (99%) | 267 (100%) | 134 (100%) | 134 (100%) | 268 (100%) |
| Mean (SD) | 8.8 (6.1) | 9.7 (6.5) | 9.2 (6.3) | 9.6 (5.8) | 11.4 (5.8) | 10.5 (5.9) |
| Median (IQR) | 9.0 (4.0, 12.0) | 9.0 (5.0, 14.0) | 9.0 (4.0, 13.0) | 9.0 (5.0, 13.0) | 11.0 (7.0, 15.0) | 10.0 (6.0, 14.0) |
| **RCADS panic disorder score** |  |  |  |  |  |  |
| N (%) | 134 (100%) | 133 (99%) | 267 (100%) | 133 (99%) | 134 (100%) | 267 (100%) |
| Mean (SD) | 6.6 (5.4) | 7.8 (6.7) | 7.2 (6.1) | 6.1 (5.0) | 7.7 (5.9) | 6.9 (5.5) |
| Median (IQR) | 6.0 (2.0, 10.0) | 6.0 (3.0, 11.0) | 6.0 (2.0, 10.0) | 5.0 (2.0, 8.0) | 6.0 (3.0, 10.0) | 5.0 (3.0, 10.0) |
| **RCADS depression score** |  |  |  |  |  |  |
| N (%) | 134 (100%) | 133 (99%) | 267 (100%) | 134 (100%) | 134 (100%) | 268 (100%) |
| Mean (SD) | 7.8 (5.6) | 8.3 (5.7) | 8.0 (5.7) | 8.0 (5.5) | 8.8 (5.8) | 8.4 (5.6) |
| Median (IQR) | 7.0 (3.0, 11.0) | 7.0 (4.0, 12.0) | 7.0 (4.0, 11.0) | 7.0 (4.0, 11.0) | 8.0 (5.0, 13.0) | 7.5 (4.0, 12.0) |
| **RCADS separation anxiety score** |  |  |  |  |  |  |
| N (%) | 134 (100%) | 133 (99%) | 267 (100%) | 134 (100%) | 134 (100%) | 268 (100%) |
| Mean (SD) | 5.8 (4.7) | 6.0 (4.4) | 5.9 (4.5) | 6.4 (4.9) | 7.3 (5.2) | 6.9 (5.1) |
| Median (IQR) | 5.0 (2.0, 9.0) | 5.0 (2.0, 9.0) | 5.0 (2.0, 9.0) | 5.0 (2.0, 10.0) | 6.0 (3.0, 12.0) | 6.0 (3.0, 10.0) |
| **RCADS generalised anxiety score** |  |  |  |  |  |  |
| N (%) | 134 (100%) | 133 (99%) | 267 (100%) | 133 (99%) | 134 (100%) | 267 (100%) |
| Mean (SD) | 6.4 (4.2) | 7.1 (4.4) | 6.7 (4.3) | 6.8 (4.3) | 7.7 (4.5) | 7.2 (4.4) |
| Median (IQR) | 6.0 (3.0, 9.0) | 7.0 (4.0, 10.0) | 6.0 (3.0, 9.0) | 6.0 (3.0, 10.0) | 7.0 (4.0, 11.0) | 7.0 (4.0, 10.0) |
| **RCADS obsessive compulsive score** |  |  |  |  |  |  |
| N (%) | 134 (100%) | 133 (99%) | 267 (100%) | 132 (99%) | 134 (100%) | 266 (99%) |
| Mean (SD) | 4.4 (3.8) | 5.0 (4.2) | 4.7 (4.0) | 3.6 (3.8) | 3.9 (3.1) | 3.7 (3.5) |
| Median (IQR) | 4.0 (1.0, 7.0) | 4.0 (2.0, 7.0) | 4.0 (2.0, 7.0) | 2.0 (0.5, 6.0) | 3.0 (2.0, 6.0) | 3.0 (1.0, 6.0) |
| **RCADS total anxiety score** |  |  |  |  |  |  |
| N (%) | 134 (100%) | 133 (99%) | 267 (100%) | 132 (99%) | 134 (100%) | 266 (99%) |
| Mean (SD) | 32.0 (19.1) | 35.6 (21.8) | 33.8 (20.5) | 32.4 (19.1) | 37.9 (20.3) | 35.2 (19.9) |
| Median (IQR) | 28.5 (16.0, 47.0) | 31.0 (19.0, 52.0) | 30.0 (17.0, 49.0) | 29.0 (17.5, 45.0) | 35.0 (23.0, 51.0) | 32.0 (19.0, 50.0) |
|  |  |  |  |  |  |  |
|  |  |  |  |  |  |  |

**Table S3.** *Comparison of mean six-month secondary assessments by treatment group (N=197)*

|  | **Treatment group** | | |  |  |  |
| --- | --- | --- | --- | --- | --- | --- |
|  | **CBT** |  | **OST** |  | **Adjusted*** |  |
| **Outcome** | **N** | **Mean (SD)** | **N** | **Mean (SD)** | **mean difference** | **95% CI** |
|  |  |  |  |  |  |  |
| SUDS before | 74 | 3.8 (3.0) | 72 | 3.7 (2.7) | 0 | -0.8 to 0.8 |
| SUDS change | 74 | -0.8 (2.1) | 70 | -0.5 (2.0) | -0.2 | -1.0 to 0.5 |
| ADIS CSR | 97 | 5.1 (2.6) | 100 | 4.9 (2.7) | 0.2 | -0.6 to 1.1 |
| CAIS child | 93 | 12.0 (11.2) | 96 | 14.5 (14.3) | -1.9 | -4.8 to 1.1 |
| CAIS parent | 92 | 14.4 (14.5) | 92 | 15.0 (15.8) | 1.9 | -1.1 to 4.9 |
| CHU-9D | 95 | 0.89 (0.10) | 96 | 0.87 (0.10) | 0.01 | -0.02 to 0.03 |
| EQ-5D-Y | 96 | 0.81 (0.24) | 98 | 0.82 (0.23) | 0 | -0.07 to 0.06 |
| EQ-5D-Y VAS | 96 | 84.2 (16.3) | 98 | 82.6 (17.1) | 1.3 | -2.8 to 5.3 |
| Goal based outcome - goal 1 | 94 | 6.4 (3.2) | 95 | 5.8 (3.4) | 0.8 | -0.0 to 1.6 |
| Goal based outcome - goal 2 | 91 | 6.2 (3.5) | 94 | 5.9 (3.2) | 0.3 | -0.6 to 1.3 |
| Goal based outcome - goal 3 | 66 | 6.5 (3.7) | 69 | 6.1 (3.2) | 0.2 | -0.9 to 1.2 |
| RCADS child social phobia score | 96 | 7.3 (5.8) | 97 | 8.6 (6.0) | -0.3 | -1.4 to 0.8 |
| RCADS child panic disorder score | 96 | 5.0 (4.7) | 97 | 5.6 (5.2) | -0.3 | -1.4 to 0.8 |
| RCADS child depression score | 96 | 5.8 (5.0) | 97 | 7.0 (4.9) | -0.8 | -1.7 to 0.2 |
| RCADS child separation anxiety score | 96 | 4.0 (4.3) | 96 | 4.2 (4.0) | -0.2 | -1.1 to 0.7 |
| RCADS child generalised anxiety score | 96 | 5.1 (3.9) | 97 | 5.2 (4.1) | 0 | -0.8 to 0.8 |
| RCADS child obsessive compulsive score | 96 | 3.5 (3.6) | 97 | 3.7 (3.9) | 0.3 | -0.5 to 1.0 |
| RCADS child total anxiety score | 96 | 24.9 (19.0) | 96 | 27.6 (19.8) | -0.5 | -4.4 to 3.4 |
| RCADS parent social phobia score | 96 | 8.5 (5.9) | 97 | 9.2 (5.6) | 0.7 | -0.4 to 1.8 |
| RCADS parent panic disorder score | 96 | 4.5 (4.1) | 97 | 5.3 (4.5) | 0.2 | -0.7 to 1.0 |
| RCADS parent depression score | 96 | 6.1 (4.9) | 97 | 6.3 (5.2) | 0.4 | -0.7 to 1.5 |
| RCADS parent separation anxiety score | 96 | 5.1 (4.8) | 97 | 5.6 (4.8) | -0.1 | -0.9 to 0.8 |
| RCADS parent generalised anxiety score | 96 | 5.2 (3.8) | 97 | 5.4 (3.8) | 0.4 | -0.3 to 1.2 |
| RCADS parent obsessive compulsive score | 96 | 2.5 (3.1) | 97 | 2.9 (3.0) | 0 | -0.7 to 0.7 |
| RCADS parent total anxiety score | 96 | 25.8 (18.1) | 97 | 28.3 (18.4) | 1.5 | -2.0 to 5.0 |

SUDS is measured on a 0-8 scale, higher scores mean more anxiety/fear

ADIS CSR is measured on a 0-8 scale higher score means phobia is more disturbing

CAIS measures impact of anxiety symptoms on psychological function on the 0-81 scale, higher scores represent greater impact

CHU-9D is measured on a 0.33-1 scale higher scores mean greater health related quality of life

EQ-5D-Y is measured on a scale of -0.594 to one (full health)

Goal based outcome is measured from 0 (goal not at all met) to 10 (goal reached)

RCADS measures anxiety and depression symptoms, higher scores mean more anxiety/depression symptoms RCADS total anxiety is measured on 0-111 scale

* adjusted for age, site, baseline ADIS CSR and baseline value of the measure as fixed effects and therapist as a random effect
